# Puerarin dry powder inhaler formulations for pulmonary delivery: Development and characterization

**DOI:** 10.1371/journal.pone.0249683

**Published:** 2021-04-13

**Authors:** Md Abdur Rashid, Saiqa Muneer, Tony Wang, Yahya Alhamhoom, Llew Rintoul, Emad L. Izake, Nazrul Islam

**Affiliations:** 1 Department of Pharmaceutics, School of Pharmacy, King Khalid University, Guraiger, Abha, Kingdom of Saudi Arabia; 2 School of Chemistry and Physics, Science and Engineering Faculty, Queensland University of Technology, Brisbane, Queensland, Australia; 3 School of Chemistry and Molecular Biosciences, Faculty of Science, University of Queensland, Brisbane, Australia; 4 Central Analytical Research Facility, Institution for Future Environment, Queensland University of Technology, Brisbane, Queensland, Australia; 5 Queensland University of Technology, Pharmacy Discipline, School of Clinical Sciences, Faculty of Health, Brisbane, Queensland, Australia; 6 Tier 2 Research Centre, Centre for Immunology and Infection, Queensland University of Technology, Brisbane, Queensland, Australia; St. John’s University, UNITED STATES

## Abstract

This study aims at developing and characterizing the puerarin dry powder inhaler (DPI) formulations for pulmonary delivery. The inhalable particles size (<2 μm) was accomplished by micronization and its morphology was examined by scanning electron microscopy (SEM). The puerarin-excipient interaction in powder mixtures was analyzed by using Fourier transform infrared spectroscopy (FTIR), Raman confocal microscopy, X-Ray powder Diffraction (XRD), and differential scanning calorimetry (DSC) methods. Using a Twin stage impinger (TSI), the in-vitro aerosolization of the powder formulations was carried out at a flow rate of 60 L/min and the drug was quantified by employing a validated HPLC method. No significant interactions between the drug and the excipients were observed in the powder formulations. The fine particle fraction (FPF) of the drug alone was 4.2% which has increased five to six-fold for the formulations with aerosolization enhancers. Formulation containing lactose as large carriers produced 32.7% FPF, which further increased with the addition of dispersibility enhancers, leucine and magnesium stearate (40.8% and 41.2%, respectively). The Raman and FTIR techniques are very useful tool for understanding structural integrity and stability of the puerarin in the powder formulations. The puerarin was found to be compatible with the excipients used and the developed DPI formulation may be considered as an efficient formulation for pulmonary delivery for the management of various diseases at a very low dose.

## 1. Introduction

Puerarin (4,7-dihydroxy-8 -d-glucosyl isoflavone) is a Chinese herbal medicine which is abundant in the root of Pueraria (*Radix puerariae*) [1]. It has been reported that puerarin has varieties of therapeutic benefits such as to relieve postmenopausal symptoms [2], prevent and reverse bone loss, inhibit the growth of breast cancer [3], and alleviate cardiovascular diseases, diabetes [4], and pulmonary arterial hypertension (PAH) [5]. Puerarin improves microcirculation and enlarges coronary artery, therefore enhancing the blood flow to the brain and can be used in the treatment of cerebral stroke and neurodegenerative disorders [6]. It also exhibited a preventive effect on immunological injury [7] and liver damage [8]. Moreover, puerarin can protect the gastric mucosa from stress-induced injury [9]. Details of the pharmacological effects of puerarin for the treatment of various diseases have been extensively reviewed by Zhou et al. [10].

Currently, puerarin is commercially available in the market as oral formulations, such as granules, capsules, and pellets. The poor solubility and absorption lead to a very low bioavailability (7%) of puerarin from these orally administered formulations [11] and thus requires daily high doses to get the therapeutic benefits. The currently available oral tablet preparation (0.28 g/ tablets) requires 5 tablets each time and three times a day for the treatment of diabetes and cardiovascular diseases [12]. Additionally, due to insufficient water solubility (0.46 mg/ml) [13], intravenous administration was thought to be the preferred drug delivery method [14] and therefore, the co-solvents like propylene glycol, ethylene glycol, and polyvinylpyrrolidone have been added in puerarin injectable formulations to increase the solubility. However, these co-solvents [15] cause adverse drug reactions such as vascular stimulation, fever, allergy and anaphylactic shock [16]. Due to the poor bioavailability, it is essential to administer high doses that consequently leads to severe adverse effects and constrains its clinical application [17]. A new delivery system would be significant to avoid the ongoing limitations of the currently available formulations. To overcome these problems, an alternative delivery strategy like pulmonary delivery as dry powder inhaler (DPI) formulation for systemic effect has been proposed for the treatment of various diseases like cerebral ischemic stroke, diabetes, and neurodegenerative disorders. The DPI formulations offer several advantages as compared to the other types of pulmonary drug delivery such as the drug is delivered, directly, deep into the lung where the extremely vascularized thin epithelial layer on top of the airways permits fast drug absorption from the target site owing to huge surface area (140m2). Additionally, DPI formulations provide enhanced physicochemical stability and deep pulmonary deposition using the patient’s respiratory force and are continuously explored as a potential route of the systemic drug delivery [18]. DPI formulation of drugs consisted of the micronized drug particles (<5 μm) mixed with the large carrier particles, preferably lactose to improve the flow property of the formulations as the inhalable particles are highly cohesive. The large carrier particles help improve the aerosolization of drug particles from the device, hence increasing the efficiency of the drug dispersion from the formulation. Lactose is the commonly used carrier by improving the flowability of powder, thus improving dose accuracy, minimizing dose variability as compared to the drug alone [19]. Leucine [20] and Magnesium stearate (Mg-St) [21] are used as dispersibility enhancers thereby enhancing the flow properties of the drug [22].

The compatibility of excipients (lactose, leucine, Mg-St, etc) with the drug used in the DPI formulation is important to ensure the stability and efficiency of the formulation. Recently, Rashid et al. demonstrated the application of Raman spectroscopy, a non-destructive analytical method in observing the distribution of all components in powder mixtures to investigate the interactions and distribution of the active drug with other excipients in the inhaled powder formulations [21]. The FTIR, another non-destructive technique in investigating the stability of active drugs in the powder mixture [23] has also been used to understand the structural integrity of puerarin in the mixture. In this study, we investigated the interactions between puerarin and some commonly used excipients in developing an efficient DPI formulation. In this study, we investigated the interactions between puerarin and some commonly used excipients and aimed to develop a DPI formulation of this drug that can achieve the required therapeutic activity at a very low dose.

## 2. Materials and methods

### 2.1 Materials

**Fig 1:**
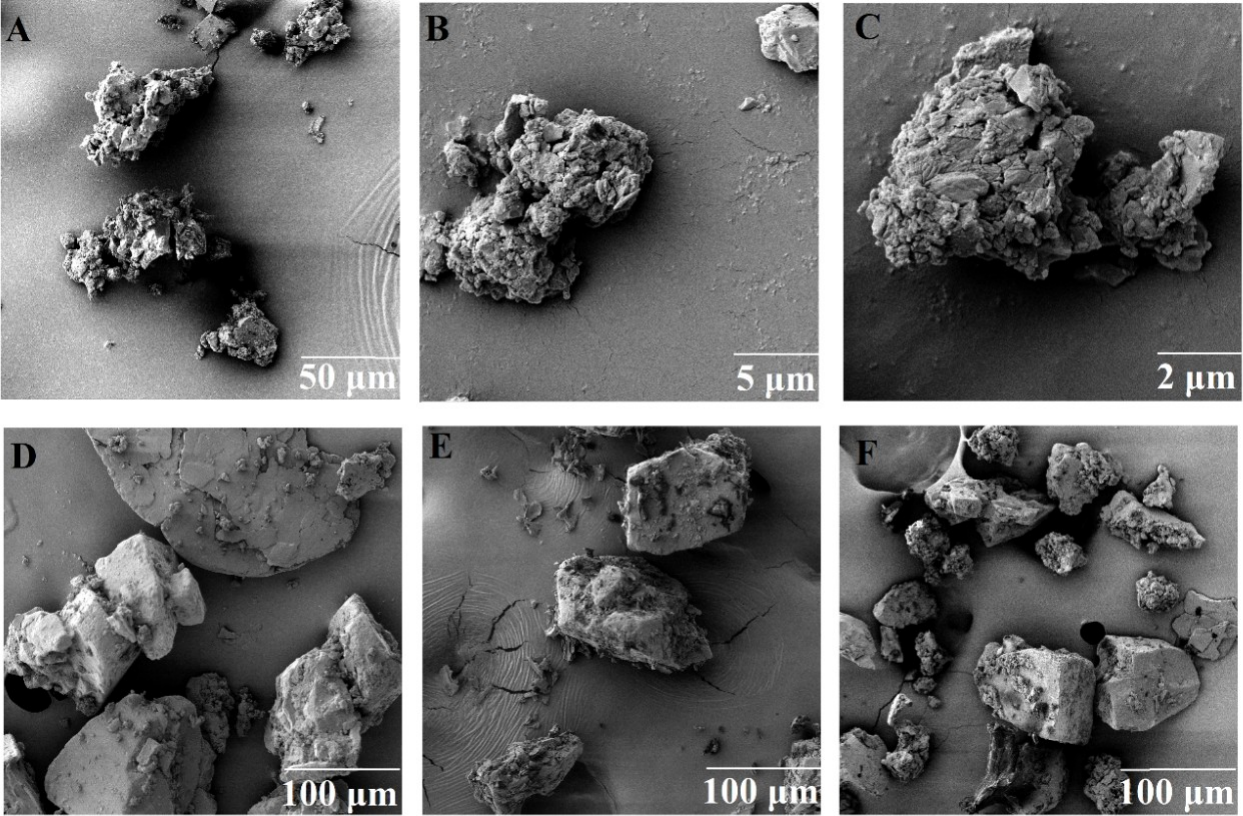
SEM images of puerarin micronized particles at different magnification: (A) Puerarin particles before crushing (original), (B) & (C) Puerarin particles before crushing; and the formulation mixtures (D) Puerarin plus Lactose (F1), (E) Puerarin plus leucine plus Lactose (F2), (F) Puerarin plus magnesium stearate plus Lactose (F3).

Puerarin (analytical grade, Fig 1), CAS Number 3681-99-0 was procured from Sigma Aldrich (P5555). Lactose monohydrate (Inhalation grade; Inhalac 120) was offered by Meggle GmbH (Wasserburg, Germany). Leucine, magnesium stearate (Mg-St) and hard gelatin capsules (size 3) were purchased from Sigma-Aldrich (Australia).

### 2.2 Methods

#### 2.2.1 Preparation of inhalable puerarin particles by micronization

Using a mortar and pestle, the puerarin particles were reduced to inhalable size (0.5–2 μm) by hand milling (micronization) [21]. However, using a mortar and pestle is not the perfect method to produce inhalable drug particles for DPI formulation due to potential repeatability problems. Collectively, based on our previously published article [21] this method has been used in this study to produce the desired particle size (<5 μm). The required size measurement was analyzed by scanning electron microscopy (SEM). The particle size was evaluated using Image J software coupled with the SEM.

#### 2.2.2 Formulation development and homogeneity test

Collaborative powder mixtures of puerarin (2.5 wt.%), leucine (5 wt.%), Mg-St (5 wt.%), and lactose (Inhalac 120; volume median diameter 157 μm) were prepared (Table 1) by validated hand mixing method [24]. Briefly, the required amount of drug particles were placed between two layers of lactose carriers with other excipients in a glass test tube. Three ceramic beads of approximately 10 mm size were placed in the tube, stoppered and the test tubes were vigorously shaken by hand for 10 minutes. The ceramic beads provided a ball-milling effect for deagglomerating the powder agglomerates that formed during mixing [24]. A batch of 2.5 g formulation of each sample was prepared in this method. The mean puerarin content in the formulations assessed the homogeneity of the drug in each mixture as presented in Table 1. Ten samples, each of around 35 mg weight, were dissolved in 100 mL of an appropriate volume of 50% ethanol (HPLC grade) and the amount of the active drug was determined by a validated HPLC method. The acceptable degree of homogeneity was achieved with a mean drug content within 100 ± 5% (mean ± SD) of the theoretical value and coefficient of variation (CV) less than 5% for all formulations.

**Table 1:** Puerarin dry powder inhaler (DPI) formulations and their dispersion behaviour.

Formulation mixtures	RD (%)	ED (%)	FPF (%)	FPD (μg)
Puerarin only	17 ± 0.4	11 ± 0.7	4.2 ± 0.1	0.27 ± 0.0
Puerarin (2.5%) + lactose (F1)	98.9 ± 0.5	67.1 ± 2.3	32.7 ± 1.5	222 ± 0.2
Puerarin (2.5%) + 5% leucine plus lactose (F2)	99.8 ± 0.5	69.9 ± 1.9	40.9 ± 0.6	286.34 ± 0.1
Puerarin (2.5%) + 5% magnesium stearate plus lactose (F3)	101 ± 1.14	65.4 ± 0.4	41.9 ± 0.8	270.9 ± 0.1

#### 2.2.3 Morphological studies by SEM

The morphology of all formulations was studied by SEM (Jeol JSM-6360A, Japan). A small quantity of dried powder was dusted onto a silicon wafer adhered on an aluminium stub over two-sided carbon adhesive tape. The stubs were air-dried and coated with a conductive layer by sputtering gold (Leica, argon gas pressure of 0.5 mbar, current of 30 mA; coating time 75 s), leading to collecting secondary electron images under high vacuum. The accelerating voltage was 5 kV and the working distance was 7.9 mm [25].

#### 2.2.4 Differential Scanning Calorimetry (DSC)

The thermal properties of all formulations were established by DSC Q100 TA Q series (TA Instruments Inc., New Castle, DE, USA). A small amount of sample was enclosed in a hermetically sealed aluminum pan, the scanning temperature was 20 to 250°C at a heating rate of 10°C/min. The stability of puerarin in different formulations was analyzed using the distinctive major DSC peaks.

#### 2.2.5 ATR-FTIR

The ATR-FTIR spectra of all puerarin mixtures were acquired using a Thermo Nicolet Nexus 870 FTIR spectrometer (Triad Scientific Inc., Manasquan, NJ, USA) equipped with a deuterated triglycine sulfate (DTGS) detector and a single reflection diamond crystal attenuated total reflectance (ATR) accessory. The angle of incidence was 40°. A minor quantity of sample was put on the top of the diamond crystal and locked with a high-pressure clamp.

#### 2.2.6 Raman spectroscopy

The spectral analysis of the individual components and the formulations of puerarin with other excipients was done using a Raman microscope, WITec Alpha 300 series (Ulm, Germany) equipped with a 532 nm laser. Initially, the spectral analysis of the individual components (active drug and all excipients) was determined. Then the spectra of the powder mixtures were taken and finally the individual spectra were mapped with the relevant spectra of the ingredients in the formulation. The Raman mapping was achieved by raster mode in a flat plane with 2 μm rise over 60×60 area of the mixture. The focal plane of the microscope was set beneath the coverslip and the laser power of 10 mW was used to record the spectra with an integration time of 1 sec. A confocal sample volume was made by the objective Zeiss 50x with 0.7NA estimating a cylinder (diameter 0.5 μm; height 3 μm). Data analysis was done by WITec Control Four and Project 4.0 software (WITec, Germany).

#### 2.2.7 X-ray powder diffraction

The drug mixtures (Table 1) and all the pure phase ingredients (puerarin, lactose, leucine and Mg-St) were analyzed for X-ray powder diffraction patterns and gathered in Debye-Scherrer geometry (capillary transmission mode) using Rigaku^®^ SmartLab furnished with CuKα (λ = 1.5418 Å) sealed X-ray tube operated at 40 kV and 40 mA. Around 35 mg of each powder sample was packed into Φ1 mm quartz capillary, which was twirled at 15 rpm for collecting data. An elliptical primary mirror in CBO-E module focused on the X-ray beam to the Hypix3000 detector working in 1D mode covering 3.82°2θ, simultaneously suppressed the CuKβ photons and white radiation background. The primary and secondary goniometer radii were kept at 300 mm. Axial aberration was reduced by using two 2.5° Soller slits. A 1 mm incident slit illuminated the capillary whose length was controlled by a 17 mm height limiting mask. On the secondary side, a 6.6 mm anti-scattering slit was located 50 mm away from the capillaries and a 12 mm anti-scattering slit was set 185 mm from the capillary. In this setup, the X-ray beam examines around 10 mg of powder samples. The X-ray diffraction patterns were accumulated from 2 to 100° 2θ and a step size of 0.02° at a scan speed of 2.0°/min.

#### 2.2.8 In-vitro aerosolization study

The formulation mixtures were filled (~25 mg) into the capsules (size 3) manually. The powder formulations were delivered through a Breezahaler^®^ (Allen and Hanburys, Middlesex, UK) and the in-vitro aerosol deposition of the powders was determined using a Twin-Stage Impinger (TSI, Apparatus, A; British Pharmacopoeia, 2000) (Copley, UK). The airflow was pulled over the TSI by vacuum pump, and the airflow rate was adjusted to 60 ± 5 L/min at the mouthpiece before each measurement [26]. The cut-off diameter for S1 is 6.5μm at 60L/min. Following each experiment, the Breezahaler® (includes capsule shells also), S1 and S2 compartments were rinsed individually with the solvent (50% ethanol), and drug particles deposited in different stages of TSI were measured using high performance liquid chromatographic method (HPLC-UV) [27]. The puerarin particle deposition from different formulations was assessed based on three parameters: the recovered dose (RD), the emitted dose (ED) and the fine particle fraction (FPF). The RD is the total amount of the drug accumulated in the Breezahaler^®^ (device, mouthpiece and capsule), S1 and S2 compartments. The ED is the percentage of RD delivered from the Breezahaler^®^. The FPF is described as the percentage of the RD deposited in the stage (S2) of the TSI [28]. The fine particle dose (FPD), the mass of puerarin deposited in the stage-2 of TSI was also determined (Table 1).

#### 2.2.9 Quantitative determination of puerarin by HPLC method

The quantitative determination of puerarin was achieved by using a modified HPLC method [29]. The analysis of puerarin was conducted by Agilent HPLC Series 1100 with Column heater and Fluorescence Detector (Heracles) Hewlett-Packard (Waldbronn, Germany). Phenomenex (Kinetex 5μ XB–C18 100A) column (250 × 4.60 mm) was used at an injection volume of 20 μL [25] and the mobile phase was composed of methanol and 0.02 M potassium dihydrogen phosphate (2.72 g of KH_2_PO_4_ was dissolved in 1 L deionized water) at a volume ratio of 30:70 at a flow rate of 1.0 mL/min [30, 31]. The detection wavelength was set at 250 nm. A calibration curve was plotted within a concentration range from 0.4 μg/ml to 50 μg/ml and the limit of quantification (LOQ) was 0.4 μg/ml. All the prepared samples were filtered before use. The HPLC was operated using Value Solution ChemStation software.

## 3. Results and discussion

### 3.1 Particle size and homogeneity of puerarin powder formulations

Micronization (pestle and mortar) was used to achieve the particle size [21, 22] and SEM was used to confirm the particle size in the range of 0.5–2 μm, which is suitable for preparing the inhaled formulations. All the formulation batches were uniformly dispersed (homogenous), having an accuracy of more than 99% and the coefficient of variation was less than 1%.

### 3.2 Scanning Electronic Microscopy (SEM)

Fig 1 demonstrates the morphologies of puerarin particles before (Fig 1A) and after crushing (Fig 1B and 1C). The sizes of the crushed particles were between 0.5–2 μm. The particle size of puerarin was reduced to less than 2 μm and the drug agglomerates were examined under SEM [32]. The aerodynamic diameter was calculated using the equation from literature and is 1.67 ± 0.01 μm [33]. The drug particles are adhered to the surface of large lactose particles in the powder mixtures (Fig 1D). Fig 1E includes drug particles and leucine adhered on the surfaces of lactose particles. Fig 1F shows the Mg-St adjoin and adhere to the large lactose carriers [22].

### 3.3 Differential Scanning Calorimetry (DSC)

**Fig 2:**
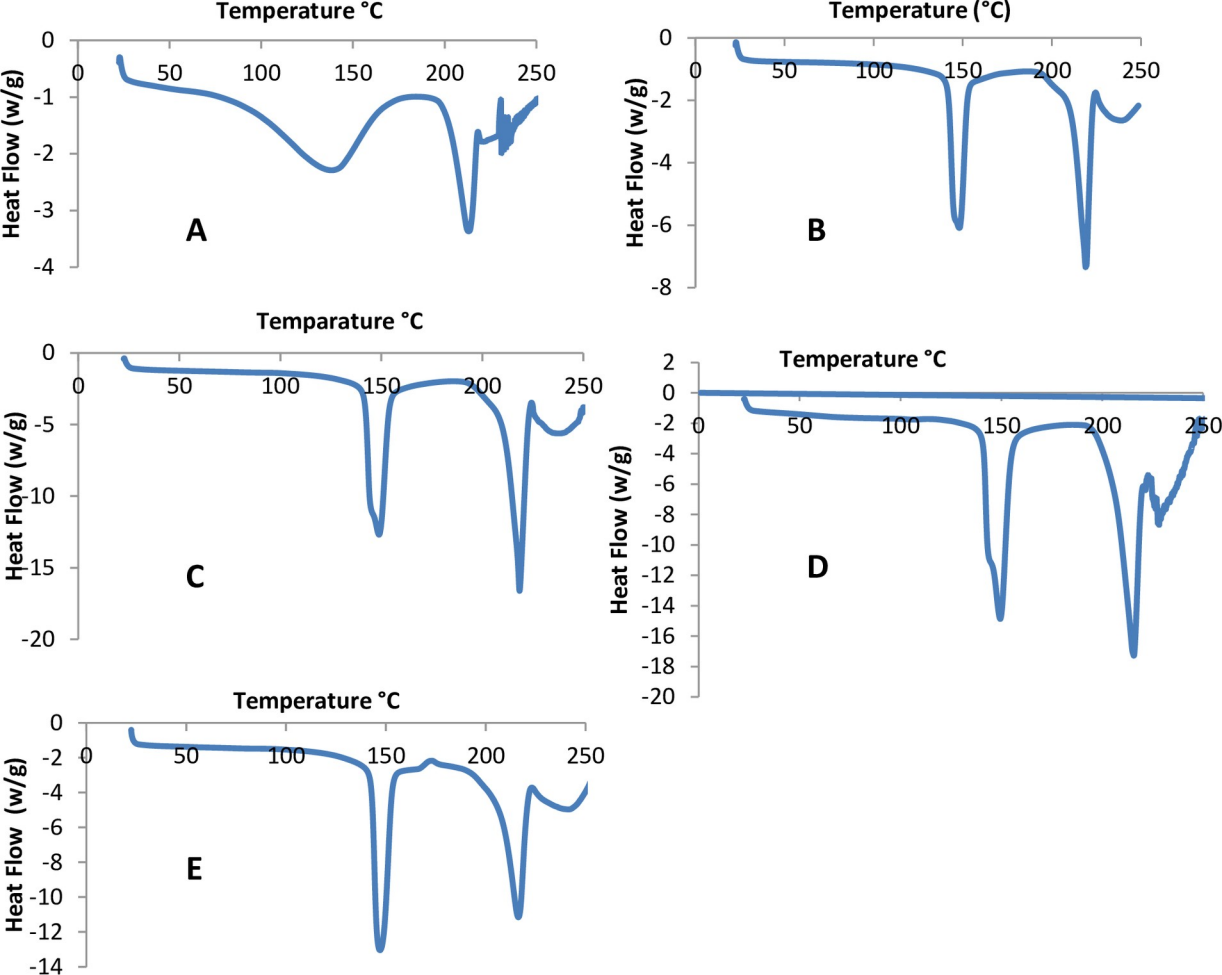
The DSC thermograms of DSC scan of Puerarin (A), Lactose (B), Puerarin and Lactose (C), Puerarin, MgSt and Lactose (D), and Puerarin, Lactose and Leucine (E).

Using DSC, the thermal behavior of Puerarin and other excipients in the formulation, was used to characterize the physicochemical property i.e, the melting point. An endothermic peak of the drug Puerarin was seen (Fig 2A) at 213.5°C corresponding to the melting point as well as a wider peak at 138.6°C, which may correspond the polymorphic form of the drug. These peaks are indicative of the polymorphs of the active drug; however, all these peaks are disappeared or merged with other excipients especially with the lactose in the formulations. This is an indication of the possibility of drug excipient interaction especially with lactose in the formulation due to the hydrogen bonding [21]. Similar thermal behaviour of another drug (glucagon) mixed with these excipients was also observed [21]. The DSC thermograms of the carrier lactose showed the presence of endothermic peaks in the range of 141–152°C (Fig 2B; a sharp peak at 148°C) that indicates the release of water of crystallization from α-lactose monohydrate. An endothermic peak in the range of 207–226°C (a sharp peak at 219°C, Fig 2B), which represents the melting of the carrier lactose crystals [34]. The mixture of the active drug, lactose and leucine showed two peaks at 215°C (215–219°C) and 147°C (147–149°C; Fig 2C–2E), which are similar to the characteristic peaks of lactose monohydrate. The reason for the slight shift of peaks could be due to the physical interactions or a lack of resolution to detect the peaks of the active drug as the amount of the active drug was very small (2.5%) in the formulations.

### 3.4 FTIR

**Fig 3:**
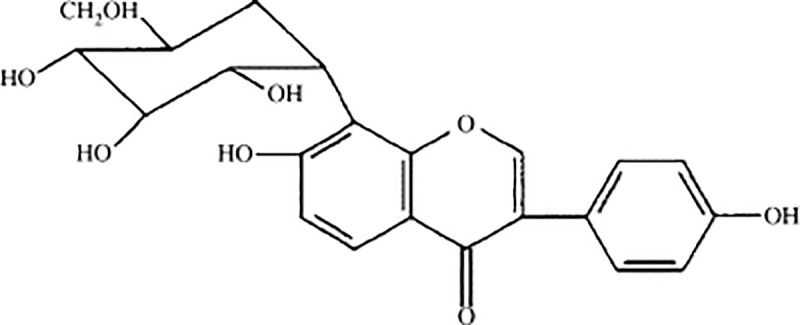
Chemical structure of puerarin.

**Fig 4:**
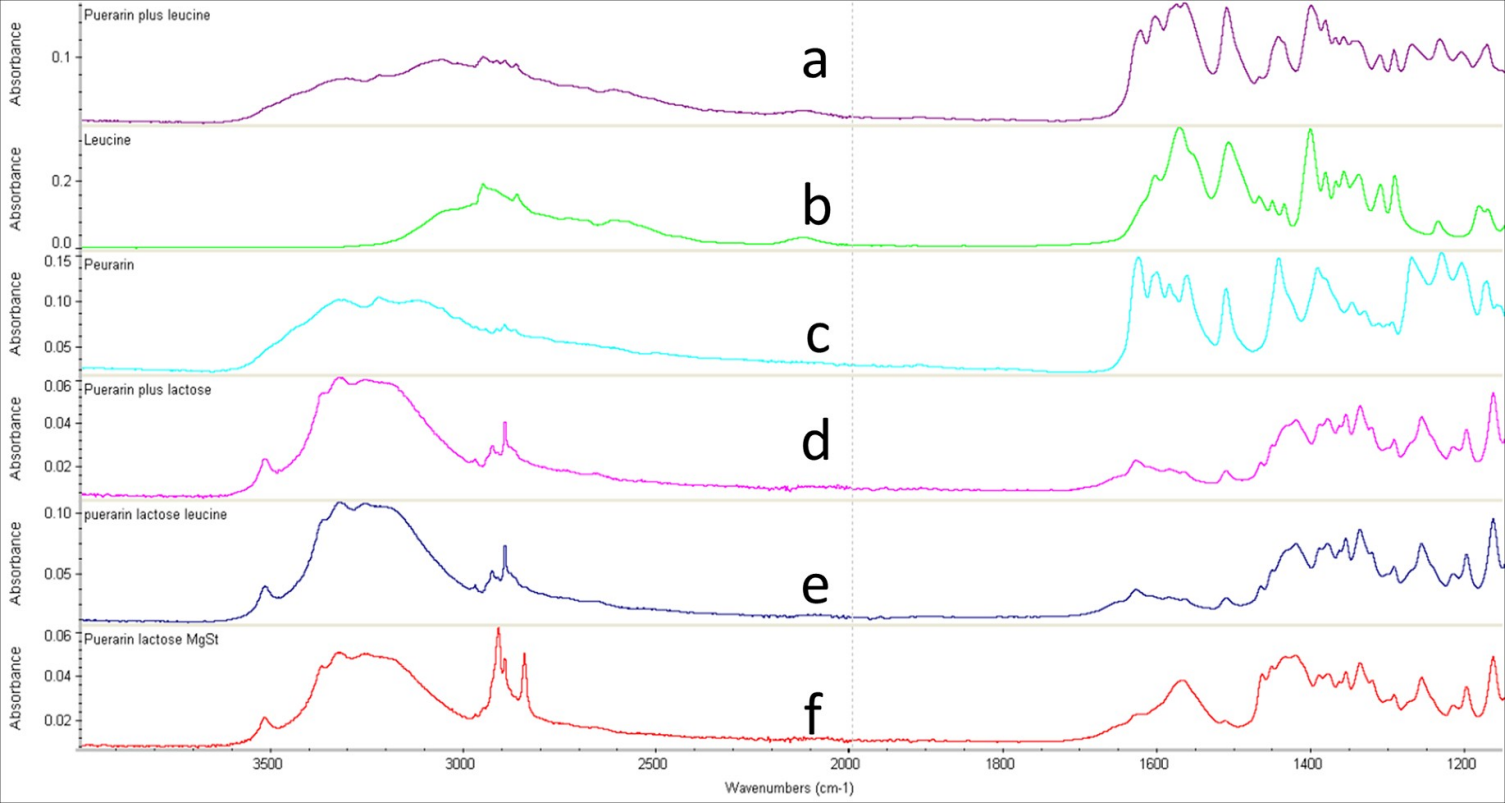
ATR-FTIR of pure puerarin plus leucine (a), Leucine (b), pure puerarin (c), puerarin plus lactose (d), puerarin plus lactose plus leucine (e), and puerarin plus Mg-St (f).

**Fig 5:**
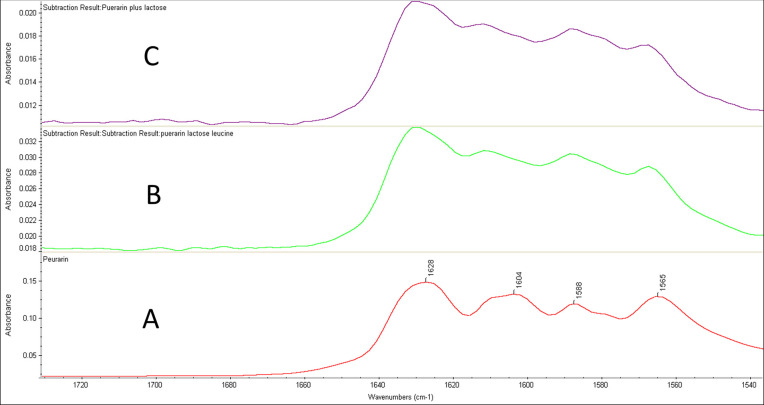
ATR-FTIR of puerarin (pure) and puerarin subtracted from the mixtures. A. Pure puerarin, B. puerarin spectra subtracted from the mixture of lactose and leucine, C. Puerarin spectra subtracted from the mixture of puerarin and lactose.

The FTIR analysis was carried out to evaluate the possible interactions between the drug and other excipients. The FTIR studies of the powder mixtures allowed for the determination of the vibration modes of the specific functional groups of the active drug (Fig 3) and the excipients used in the formulations. The FTIR spectra of puerarin and puerarin mixed with lactose, leucine and MgSt have been presented in Fig 4A–4F. The FTIR of puerarin showed clear characteristic peaks at 1628 cm^-1^, 1588 cm^-1^, and 1272 cm^-1^ which relates to the band stretching of the aldehyde group (C = O), benzene ring, and skeleton and C-O absorption peak, respectively (Fig 4A). After mixing with lactose, leucine and Mg-St, this characteristic peak for C = O has not been changed (a minor shifting 1630 cm^-1^), which indicates that the excipients in the powder mixtures did not affect the stability of the pure drug. However, puerarin peak was disappeared (a tiny shoulder peak around 1630 cm^-1^) from the spectra of the mixture of puerarin, lactose and Mg-St (Fig 4F). The reason behind this could be due to poor resolution to detect the puerarin peak in this sample. To confirm the presence or absence drug interactions in the formulations, the peak for puerarin was subtracted from the mixtures and found no remarkable changes as presented in Fig 5A–5C. A slight shifting of the puerarin peaks occurred in the mixtures due (B and C) to the interactions between puerarin and excipients especially the lactose and leucine due to the possible hydrogen bonding [19]. However, no chemical changes of the drug occurred in the formulations.

### 3.5 Raman studies

**Fig 6:**
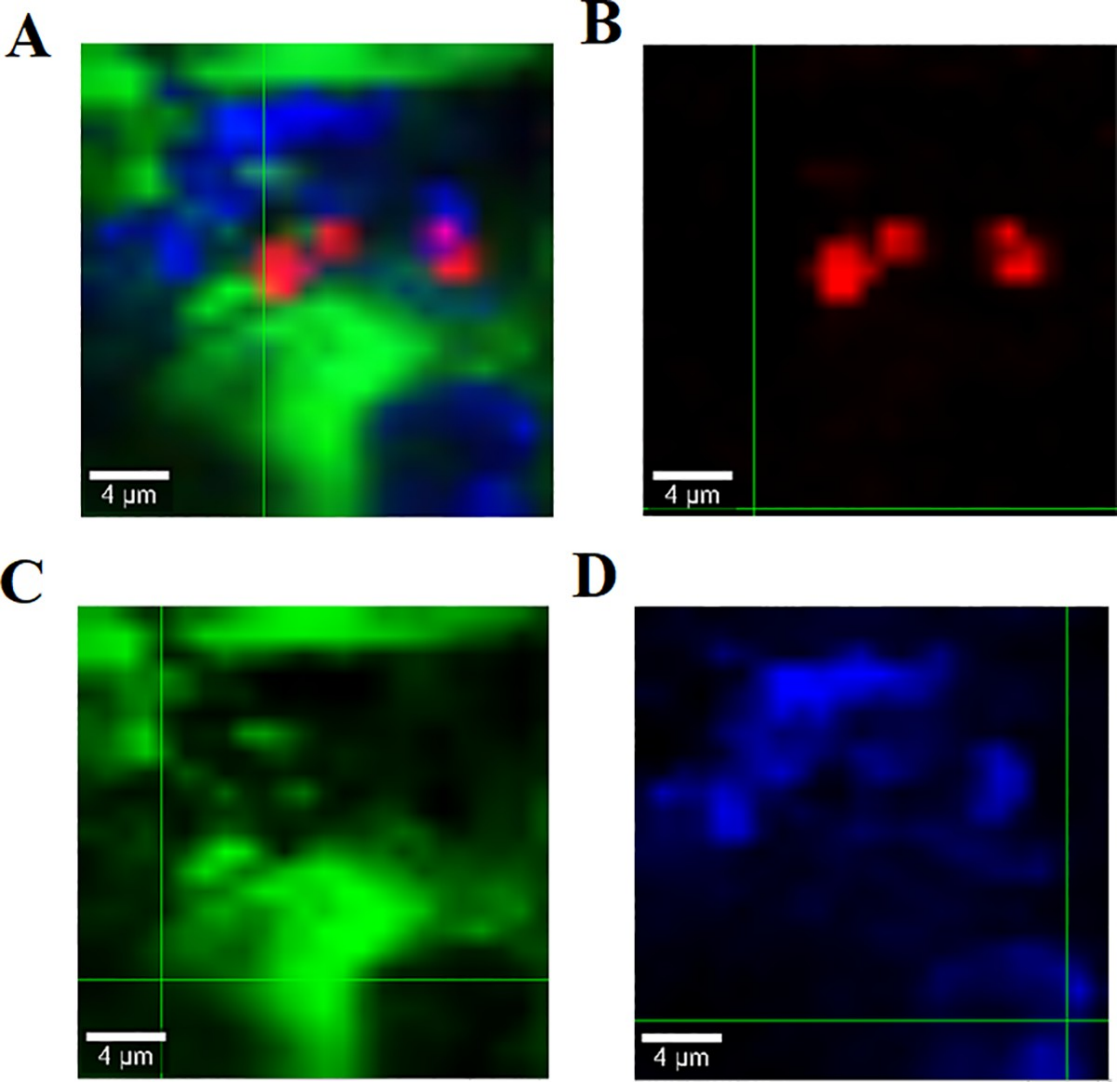
Raman mapping of formulation F3 (A) drug and excipient mixture, (B) Puerarin (C) Lactose, and (D) Mg-St.

The images in Fig 6A–6D depict the identification and distribution of individual components in formulation mixtures. The Raman mapping was done for formulation F3 with lactose, Mg-St and puerarin. In Fig 6, image A is a combination of all the components and illustrates that all are uniformly distributed in the mixture and puerarin particles are adhered on the surface of the lactose particles with Mg-St surrounding it which is expected to help the particle flow freely and detach from the carrier surfaces during inhalation [35]. Other images represent the individual components of the mixture as (B) representing Puerarin, (C) lactose, and (D) MgSt.

### 3.6 XRD crystal structure refinement and quantitative phase analysis

**Fig 7:**
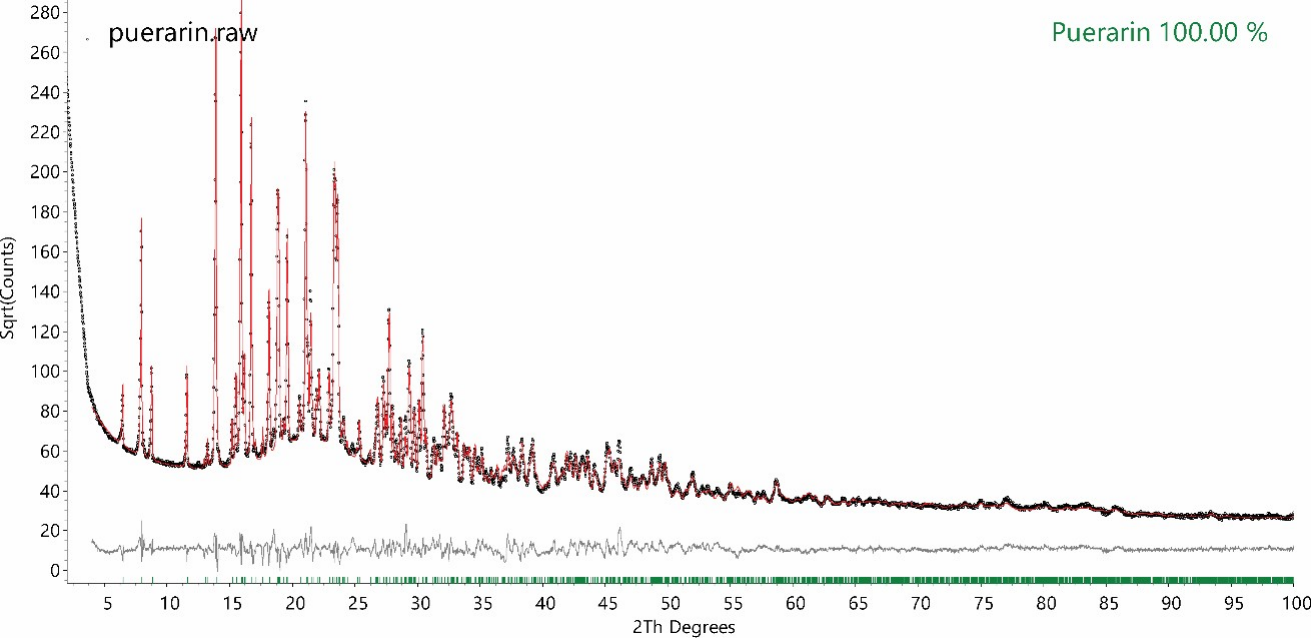
**Rietveld refinement of the puerarin crystal structure using rigid body model to simulate (red line) the measured XRD pattern (black dot) of the pure puerarin sample.** Rwp = 7.36%, χ^2^ = 3.96.

The measured XRD pattern of the pure puerarin sample and the corresponding fitting from Rietveld refinement of its crystal structure is shown in Fig 7. The unit cell of puerarin monohydrate crystals is in triclinic system (Space Group: P1), with lattice parameters a = 6.35637(9) Å, b = 11.4823(3) Å, c = 14.1401(6) Å, α = 73.967(3)° β = 88.135(2)° γ = 88.4534(14)°. Each unit cell contains two puerarin molecules with different torsion angles of its phenol groups. Each molecule carries one water molecule, though the H-bonds from water molecules were connected to different groups. One puerarin molecule has H-bond links to the hydroxyl group on its phenol group, while the other puerarin molecule has H-bond links to the carbonyl group on its flavone mother nucleus [36]. The molecular structure of puerarin was modelled using a rigid body in DIFFRAC.TOPAS v6 software [37]. The positions and orientations of the two molecules, the torsion angles of their phenol groups, of their glucose ring, and of the hydroxymethyl groups on their glucose ring were optimised during crystal structure refinement. The refined crystal structure of puerarin monohydrate is provided as.cif in the (S1 Fig in S1 File).

**Fig 8:**
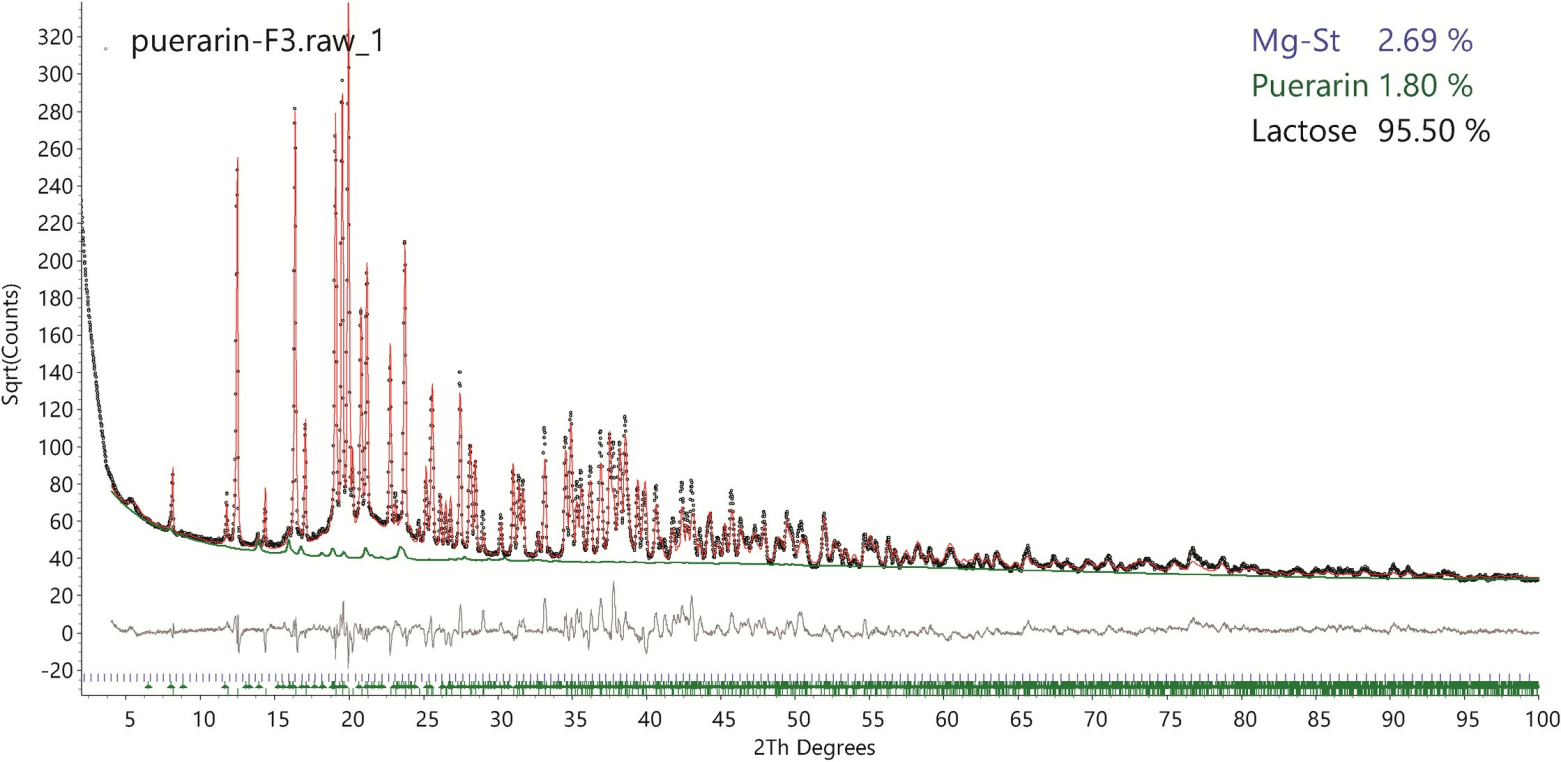
Quantitative phase analysis of the formulation F3. The weight percentage of each ingredient are shown in the upper right corner. The diffraction signal contribution from the API, puerarin, is highlighted in the green line.

Since the refined crystal structure of puerarin monohydrate achieved good fitting to its measured XRD pattern, it enables accurate quantitative phase analysis (QPA) for the API concentration, which is usually the minor phase of low concentration in the formulated mixture (Table 1). The crystal structure models of α-lactose monohydrate and leucine, as well as the calibrated PONKCS model of Mg-St that has been previously reported [22]. The QPA fitting of formula F3 (Table 1) is shown in Fig 8. The refined weight percent of puerarin and Mg-St is less than the designed value. Since normally around 10 mg of powder sample is measured in capillary transmission XRD, the hand milling preparation method [24] could lead to local in-homogeneity. The QPA fittings of formula F1 and F2 are available in the (S2 and S3 Figs in S1 File, respectively). The diffraction signal contributions of all the ingredient phases are clear without new diffraction peaks from any other phase detected in the powder mixtures. This indicates the drug puerarin and excipients show no chemical interactions in the mixtures.

### 3.7 Aerosolization of puerarin from DPI formulations

The in-vitro aerosolization was represented by the recovered (RD), emitted doses (ED), fine particle fraction (FPF) and fine particle dose (FPD) (Table 1) which was calculated using calibration plot and the corresponding linear regression equation presented in the (S4 Fig in S1 File) [38]. The ED was between 65–70% which indicate that the particles were not effectively emitted from the Breezahaler^®^ [39]. The FPF of drug only formulation was 4.2% which could be due to the cohesiveness of particles and poor flowability [21]. To overwhelm this poor flow, the micronized particles were mixed with lactose large carrier particles (Inhalac 20, size 157 μm) which increased the FPF up to 32.7%, which is 8 times higher than that of the pure drug formulation. The large carrier particles increase the FPF of formulations which is due to a decrease in the cohesive nature of the drug particles and enhance the deagglomeration of the particles due to the collision effects. To further investigate the effects of the dispersibility enhancers on FPF, leucine and Mg-St were added in formulation F2 and F3. The high FPF of the formulations F2 and F3 (containing leucine and Mg-St); 40.9% and 41.9%, respectively are the evidence of the greater dispersion and aerosolization of puerarin as compared to drug only formulation devoid of the excipients [40]. The recovered dose ranged between 98% to 100% for all the formulation mixtures [41]. Hence, it is recognized that the only lactose mixture can produce a promising FPF and RD, whereas, with the addition of other excipients like leucine and Mg-St, the amount of RD dose and ED has improved which contributed to better performance of the formulations. Hence, the excipients such as lactose, leucine and Mg-St are considered to use as appropriate excipients for the development of DPI formulations of puerarin. The highest FPD (286.3 μg) of puerarin was obtained from the formulation containing lactose with the dispersibility enhancer leucine (Table 1). Assuming that all of the drug deposited into deeps lungs will be absorbed and will be available into plasma for therapeutic actions upon pulmonary delivery. Using a rat model, it was reported that a 10 mg/kg orally administered puerarin produced the plasma concentration of 228 μg/L (0.228 μg/mL) [42]. Based on this literature, an oral administration of a high dose is required to get pharmacological action. Our in-vitro aerosolization data (FPD, Table 1) showed that 286.3 μg/100 mL (2.86 μg/mL) puerarin would be available from an inhaled dose of 875 μg of puerarin (35 mg of 2.5% puerarin mixture with excipients). This predicted plasma concentration of puerarin upon inhalation of a very low dose of the formulation is promising. Thus, the pulmonary delivery of puerarin is expected to produce a much higher plasma concentration at a very low dose of the drug when given in the form of DPI [22]. Although the FTIR and DSC data evidenced the physical interactions of puerarin with other excipients especially the lactose and leucine; however, no chemical degradation of the drug occurred as demonstrated by the determination of active drug by HPLC. Therefore, using these excipients, the developed DPI formulation of puerarin is expected to ensure the efficient drug dispersion and improve the absorption and bioavailability of the drug upon pulmonary delivery.

## 4. Conclusion

The DPI formulations of the herbal drug puerarin were successfully developed and characterized. SEM was used to study the particle size and morphology of the formulations and found to be within 0.5–2 μm range. Although the DSC and FTIR data showed physical interaction of the drug with other excipients; the Raman and XRD data revealed no interactions of the drug with the excipients used to develop the formulation. The HPLC method perfectly determined the active drug in the formulation, no chemical interactions between the drug and other excipients were occurred, which is an indication of the stability of the powder formulations. The QPA obtained from XRD analysis resulting from 10 mg sub-sampling of the formulated mixture provides the quantitative analysis of all components in the mixtures. The DSC and XRD techniques are useful tools for studying the physicochemical properties of the active drug. Both the Raman and FTIR techniques are very useful methods for understanding the stability and structural integrity of the puerarin in the powder formulations. The Raman helped understand the distribution of all components in the powder mixtures. Therefore, these excipients (lactose, leucine and Mg-St) can be used to develop an efficient puerarin DPI formulation for pulmonary delivery. The outcome of this study is promising as the FPF of the developed puerarin DPI formulations with excipients being 32–42%, which are within the range of currently available marketed DPI products. Based on the in-vitro lung deposition data (FPF and FPD, Table 1), it is expected that the developed DPI formulation would be therapeutically effective in the treatment of various diseases such as diabetes, cardiovascular, and neurodegenerative disorders at a very low dose.

## Supporting information

S1 File (PDF)
