# Supplementary material for: Puerarin dry powder inhaler formulations for pulmonary delivery: Development and characterization
Source: PLoS One. 2021 Apr 13;16(4):e0249683. doi: 10.1371/journal.pone.0249683 (PMC8043385; doi:10.1371/journal.pone.0249683)
Supplement: S1 File — (PDF) [file pone.0249683.s001.pdf]

Supplementary Information

Puerarin dry powder inhaler formulations for pulmonary delivery: development and characterization

Md Abdur Rashid [1*], Saiqa Muneer [2,3], Tony Wang [4], Yahya Alhamhoom[1], Llew Rintoul[4], Emad L Izake[2], Nazrul Islam[5,6*]

[1]Department of Pharmaceutics, School of Pharmacy, King Khalid University, Guraiger, Abha 62529, Kingdom of Saudi Arabia (mdrashid@kku.edu.sa (M.A.R.); ysalhamhoom@kku.edu.sa (Y.A).

[2]School of Chemistry and Physics, Science and Engineering Faculty, Queensland University of Technology, Brisbane, Queensland, Australia. (saiqa.muneer@hdr.qut.edu.au (S.M); e.kiriakous@qut.edu.au (ELI)

[3]School of Chemistry and Molecular Biosciences, Faculty of Science, University of Queensland, Brisbane, Australia.

[4]Central Analytical Research Facility, Institution for Future Environment, Queensland University of Technology, Brisbane, Queensland, Australia (
tony.wang@qut.edu.au (T.W); l.rintoul@qut.edu.au; L.R)

[5]Queensland University of Technology, Pharmacy Discipline, School of Clinical Sciences, Faculty of Health, Brisbane, Queensland, Australia. (nazrul.islam@qut.edu.au (N.I.)

[6]Institute of Health and Biomedical Innovation, Queensland University of Technology, Brisbane, Queensland, Australia.

*indicates the Corresponding author

Correspondence: mdrashid@kku.edu.sa (M.A.R.); nazrul.islam@qut.edu.au (N.I.);
Tel.: +966-17-241-8408 (M.A.R.); +61-07-313-81899 (N.I.)

References

1. Zhang, S., Ji, G. and Liu, J., 2006. Reversal of chemical-induced liver fibrosis in Wistar rats by puerarin. J Nutr Biochem, 17, pp.485-491.

Crystallography Information File of the puerarin monohydrate crystal structure refined in this study:

```
data_
_chemical_name_mineral 'Puerarin'
_cell_length_a 6.35637(9)
_cell_length_b 11.4823(3)
```

```
_cell_length_c 14.1401(6)
_cell_angle_alpha 73.967(3)
_cell_angle_beta 88.135(2)
_cell_angle_gamma 88.4534(14)
_cell_volume 991.19(5)
_symmetry_space_group_name_H-M P1
loop_
       _symmetry_equiv_pos_as_xyz
        'x, y, z '
loop_
_atom_site_label
_atom_site_type_symbol
_atom_site_symmetry_multiplicity
_atom_site_fract_x
_atom_site_fract_y
_atom_site_fract_z
_atom_site_occupancy
_atom_site_B_iso_or_equiv
O11 O   1 0.4322105 0.5993533 0.2241583 1 5.08482
O12 O   1 -0.1019299 0.9152843 0.3948398 1 3.632014
O13 O   1 -0.2620272 1.086756 0.7972397 1 3.718867
O14 O   1 0.2640833 0.8697798 0.6531678 1 2.984568
O15 O   1 0.007364006 0.9271584 0.8912302 1 3.063525
O16 O   1 -0.1025787 0.6945618 1.034269 1 5.448022
O17 O   1 0.2947464 0.9674108 1.09095 1 5.061133
O18 O   1 0.3129535 1.188661 0.9636576 1 4.09786
O19 O   1 0.334817 1.181645 0.7513255 1 3.110899
O31 O   1 0.5712957 0.7126033 0.8971817 1 4.658453
O32 O   1 1.035284 0.3679242 0.690846 1 3.339874
O33 O   1 1.17441 0.1417716 0.3217872 1 3.734658
O34 O   1 0.7654849 0.4117563 0.427757 1 2.850342
O35 O   1 0.7198051 0.1575565 0.2745763 1 2.921403
O36 O   1 0.9237582 -0.03553282 0.2187068 1 4.303148
O37 O   1 0.7443466 0.2440168 0.01728411 1 3.671493
O38 O   1 0.8620135 0.4555996 0.00516839 1 4.476853
O39 O   1 0.9057335 0.4773317 0.213507 1 3.616223
C1 C   1 0.40856 0.64534 0.29546 1 3.600432
C2 C   1 0.55675 0.70747 0.32965 1 3.450414
C3 C   1 0.50041 0.76728 0.39866 1 3.23723
C4 C   1 0.29639 0.76392 0.43423 1 2.874029
C5 C   1 0.14892 0.7003 0.40111 1 3.339874
C6 C   1 0.20508 0.64085 0.33184 1 3.789928
C7 C   1 0.22272 0.83429 0.5055 1 2.573993
C8 C   1 0.03471 0.91069 0.48357 1 2.637158
C9 C   1 -0.03483 0.96522 0.55943 1 2.289748
```

C10 C   1 -0.2543 1.04134 0.55082 1 3.276709

C11 C   1 -0.30746 1.08556 0.62806 1 3.063525

C12 C   1 -0.18417 1.0672 0.70749 1 2.645054

C13 C   1 0.00059 1.00403 0.71443 1 2.289748

C14 C   1 0.06863866 0.9678894 0.644313 1 2.392392

C15 C   1 0.314525 0.8239222 0.5816909 1 2.866133

C16 C   1 0.1241206 0.973035 0.8018604 1 2.566097

C17 C   1 0.2069427 1.105209 0.8252966 1 2.297644

C18 C   1 0.2656853 1.076594 0.929224 1 2.850342

C19 C   1 0.14928 0.99791 1.00973 1 3.063525

C20 C   1 0.1023467 0.8778385 0.9840729 1 3.110899

C21 C   1 -0.05887 0.80953 1.05351 1 3.995216

C31 C   1 0.63303 0.62248 0.8253 1 3.521475

C32 C   1 0.79664 0.67348 0.76637 1 3.797824

C33 C   1 0.85956 0.63072 0.68986 1 3.947842

C34 C   1 0.75895 0.53691 0.67233 1 2.787176

C35 C   1 0.59542 0.48587 0.7313 1 4.358417

C36 C   1 0.53251 0.52862 0.8078 1 4.73741

C37 C   1 0.81027 0.47071 0.58985 1 2.59768

C38 C   1 0.985 0.38489 0.60067 1 2.637158

C39 C   1 1.03665 0.33071 0.52137 1 2.495036

C40 C   1 1.21021 0.2744 0.53817 1 3.047734

C41 C   1 1.2431 0.2288 0.46054 1 3.126691

C42 C   1 1.12019 0.24377 0.38194 1 2.795072

C43 C   1 0.93488 0.30491 0.3651 1 2.495036

C44 C   1 0.9569825 0.3494156 0.4417358 1 2.392392

C45 C   1 0.7141079 0.4742101 0.5221354 1 2.952986

C46 C   1 0.8032704 0.3183807 0.2806094 1 2.621367

C47 C   1 0.8854471 0.4024769 0.1618527 1 2.637158

C48 C   1 0.8175105 0.3862141 0.09129649 1 2.881924

C49 C   1 0.7841532 0.2486451 0.1022704 1 2.700324

C50 C   1 0.7054428 0.1964187 0.1710397 1 2.818759

C51 C   1 0.7048004 0.05095374 0.1922695 1 3.647806

H2 H   1 0.69511 0.70885 0.30601 1 4.105755

H3 H   1 0.59964 0.8103 0.4217 1 3.868885

H5 H   1 0.01158 0.69802 0.42613 1 4.026799

H6 H   1 0.10477 0.59769 0.30961 1 4.579496

H10 H   1 -0.34278 1.05589 0.49749 1 3.947842

H11 H   1 -0.40387 1.15808 0.61169 1 3.710971

H15 H   1 0.44352 0.78027 0.60165 1 3.474101

H16 H   1 0.26111 0.94043 0.80132 1 3.079317

H17 H   1 0.05323 1.15016 0.83288 1 2.763489

H18 H   1 0.4265 1.04159 0.9286 1 3.395144

H19 H   1 0.01747 1.04106 1.01721 1 3.710971

H20 H   1 0.22958 0.83498 0.9814 1 3.710971

```
H32 H   1 0.86598 0.73813 0.7786 1 4.579496
H33 H   1 0.97225 0.66628 0.64957 1 4.73741
H35 H   1 0.5278 0.4212 0.71842 1 5.211151
H36 H   1 0.41999 0.49368 0.84842 1 5.684892
H40 H   1 1.30987 0.25896 0.58705 1 3.632014
H41 H   1 1.36682 0.18267 0.46568 1 3.789928
H45 H   1 0.60388 0.53422 0.50642 1 3.553058
H46 H   1 0.6493 0.34391 0.2917 1 3.158273
H47 H   1 1.03536 0.38453 0.17928 1 3.158273
H48 H   1 0.63856 0.41069 0.08811 1 3.474101
H49 H   1 0.95304 0.22897 0.09131 1 3.23723
H50 H   1 0.53822 0.2022 0.17884 1 3.395144
H111 H   1 0.57827 0.59429 0.21577 1 6.948201
H131 H   1 -0.36766 1.12725 0.78734 1 4.73741
H161 H   1 -0.15645 0.68544 0.9938 1 6.316547
H171 H   1 0.15864 0.97404 1.1453 1 6.079676
H181 H   1 0.41397 1.20091 0.97859 1 4.974281
H191 H   1 0.25937 1.24705 0.73605 1 3.553058
H211 H   1 -0.0214 0.80753 1.11995 1 4.816367
H212 H   1 -0.19651 0.848 1.03962 1 4.816367
H311 H   1 0.48814 0.61405 0.94187 1 5.763849
H331 H   1 1.1012 0.17341 0.27602 1 4.658453
H361 H   1 0.88623 -0.04472 0.30142 1 4.974281
H371 H   1 0.78619 0.26518 -0.04726 1 4.50054
H381 H   1 0.92845 0.41432 -0.027 1 4.895324
H391 H   1 0.87102 0.57395 0.12828 1 8.685252
H511 H   1 0.58539 0.00449 0.23306 1 4.342626
H512 H   1 0.69754 0.02946 0.12787 1 4.342626
O71 O   1 0.3684895 0.5899871 0.0561253 1 5.89018
O72 O   1 0.8985763 0.3604796 0.8916844 1 4.461061
H711 H   1 0.40829 0.57463 0.11211 1 7.816727
H712 H   1 0.26561 0.53586 0.05285 1 7.816727
H721 H   1 0.95706 0.38599 0.8111 1 16.42302
H722 H   1 1.07375 0.30867 0.90405 1 5.526978
```

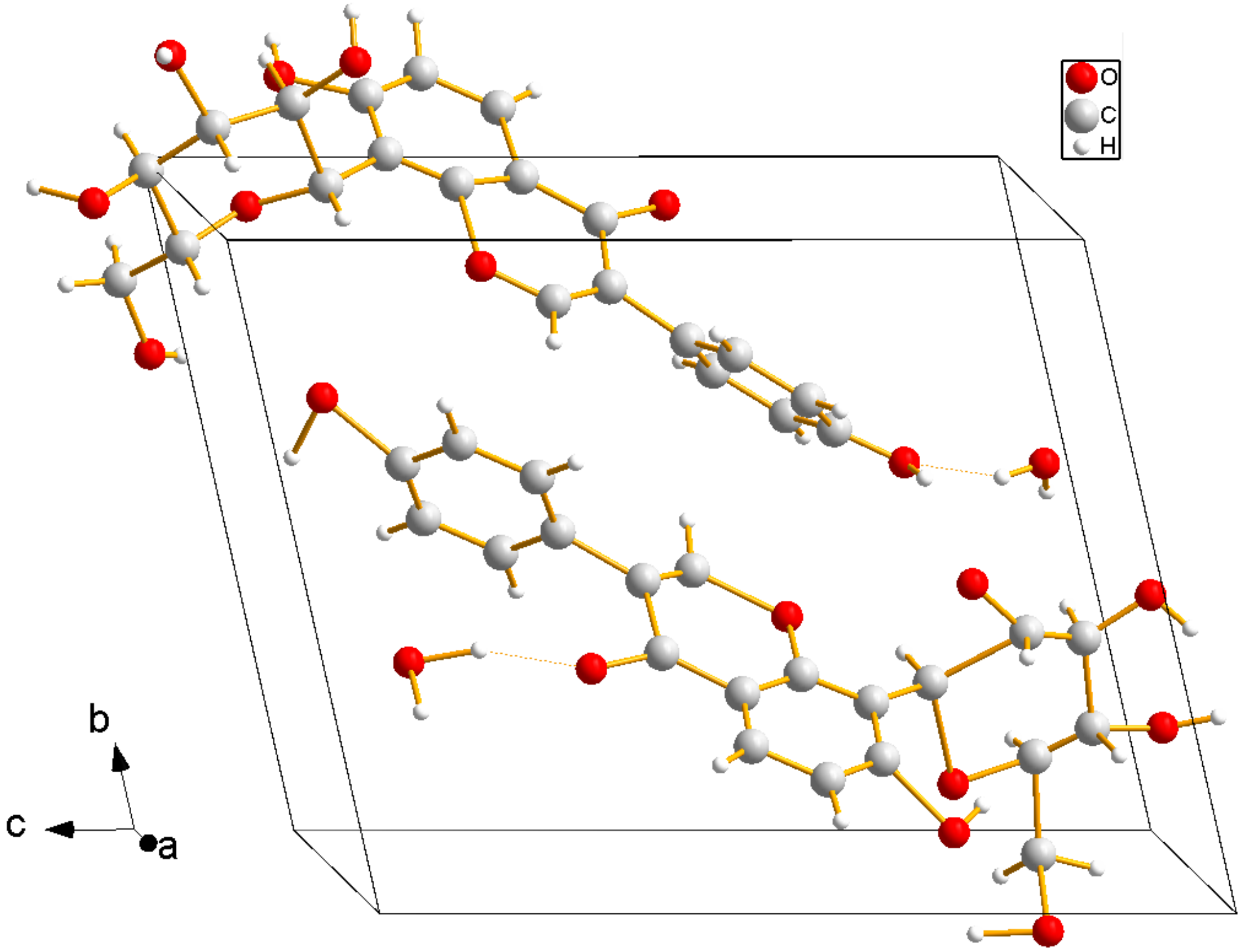

Fig. S1. The refined crystal structure of puerarin monohydrate as described in the .cif format

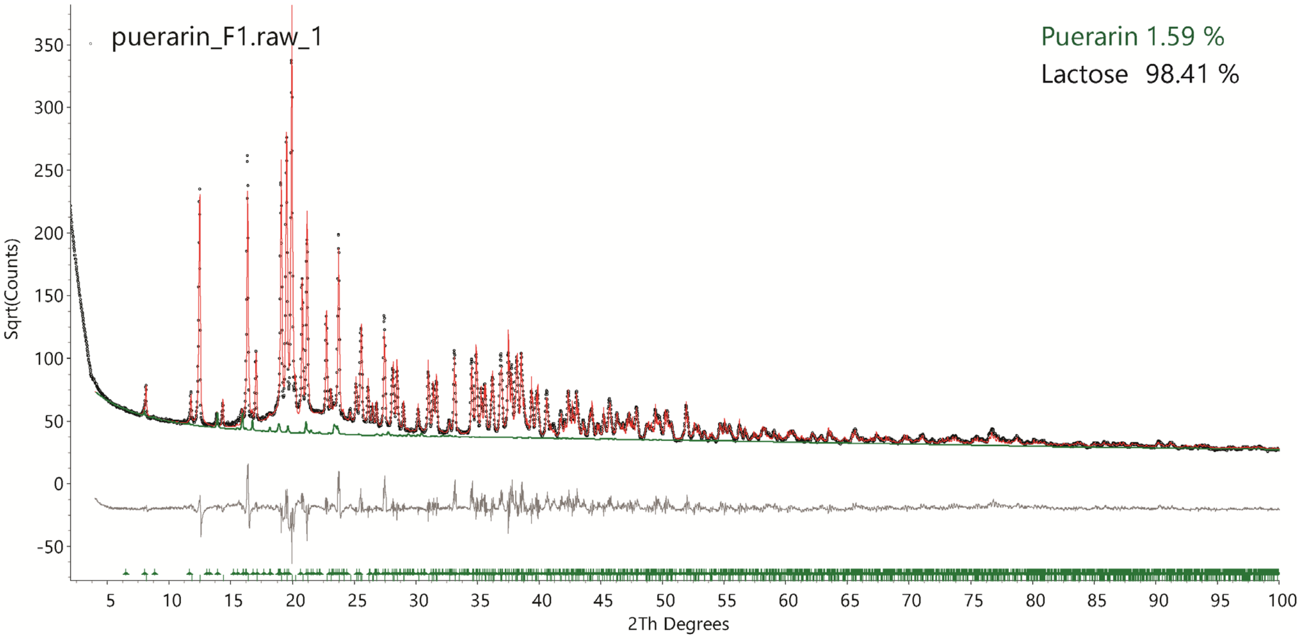

Fig. S2. Quantitative Phase Analysis of the formulation F1. The weight percentage of each ingredients are shown on the upper right corner. The diffraction signal contribution from the API, puerarin, is highlighted in green line.

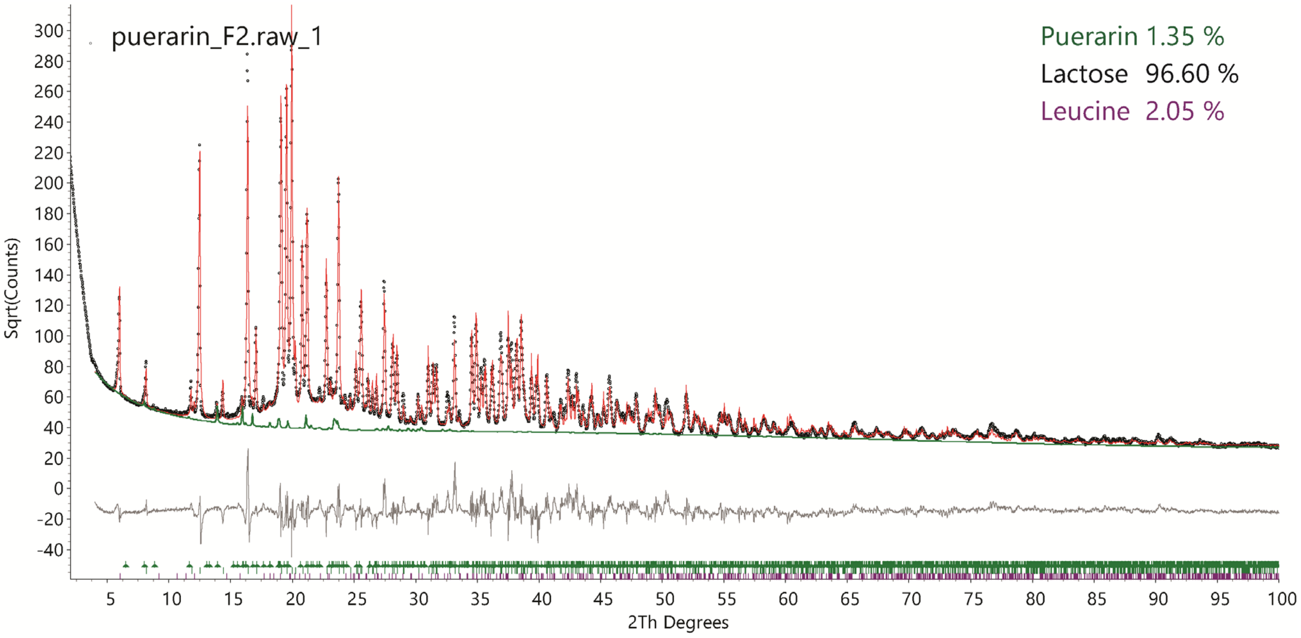

Fig. S3. Quantitative Phase Analysis of the formulation F2. The weight percentage of each ingredients are shown on the upper right corner. The diffraction signal contribution from the API, puerarin, is highlighted in green line.

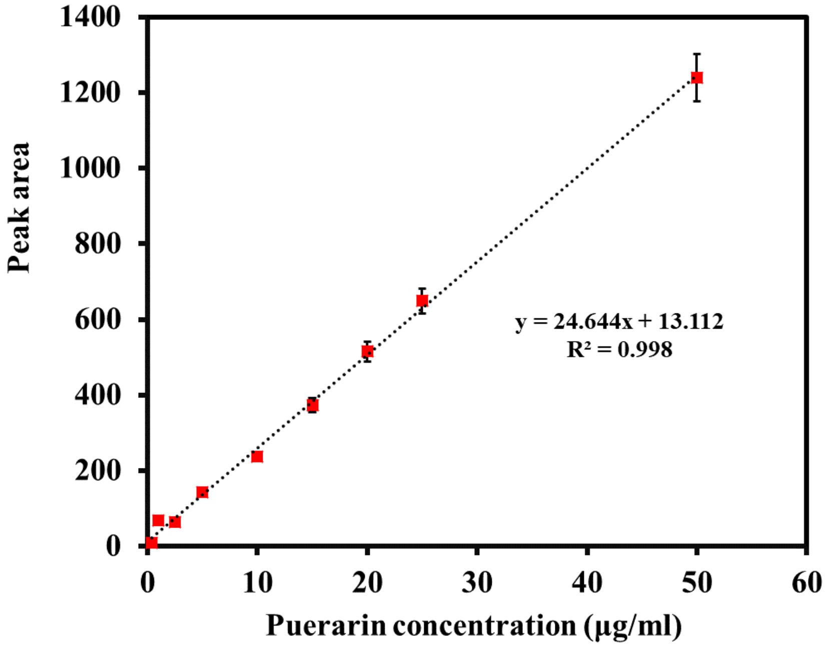

Fig. S4. Calibration plot of puerarin at concentration range of 0.4 µg/ml to 50 µg/ml.
